# Effect of Innovative Low-level Laser Therapy Protocol for Stress Urinary Incontinence: A Case Series Study: Low-level Laser Therapy for Stress Urinary Incontinence

**DOI:** 10.31661/gmj.v14i.3841

**Published:** 2025-08-27

**Authors:** Fateme Hoseinzade, Mozhgan Ayazi, Nahid Tafazoli Harandi, Michael Hans Weber, Yasaman Zandi Mehran, Shila Mirzadeh

**Affiliations:** ^1^ Physical Medicine and Rehabilitation Specialist, Isfahan University of Medical Sciences, Isfahan, Iran; ^2^ Gynecologist, Kermanshah University of Medical Science, Kermanshah, Iran; ^3^ Obstetric and Gynecologist, Infertility Fellowship, Isfahan University of Medical Sciences, Isfahan, Iran; ^4^ Head of International Society of Medical LASER Applications, Lauenfoerde, Germany; ^5^ Biomedical Engineering Center, Dubai, UAE; ^6^ Dermatologist, Shiraz University of Medical Science, Shiraz, Iran

**Keywords:** Low-level Laser Therapy, Transvaginal Laser, Intra-bladder Laser, Stress Urinary Incontinence

## Abstract

Stress urinary incontinence (SUI) is a prevalent condition characterized by involuntary urine leakage during physical activities. This condition can significantly impact a woman’s quality of life. Treatment options for SUI include behavioral therapy, medication, and surgery. However, non-pharmacological methods such as low-level laser therapy (LLT) have demonstrated the potential to promote tissue repair, reduce inflammation, and improve pelvic floor muscle function in various pelvic floor disorders. Despite these benefits, the effectiveness of combined transvaginal and intra-bladder LLT for managing SUI has not been extensively studied. This case series aimed to assess the safety and efficacy of a novel regimen using concurrent transvaginal and intra-bladder LLT for the treatment of stress urinary incontinence in perimenopausal and postmenopausal women. The results appear promising, and further investigation is recommended. Trial Registration: This clinical trial was registered in the Iranian Registry of Clinical Trials (IRCT) under the registration number IRCT20231203060255N2, on 30 June 2024 (retrospectively registered).

## Introduction

Stress urinary incontinence (SUI) is defined as the involuntary leakage of urine during activities that increase intra-abdominal pressure—such as exercise, sneezing, coughing, or laughing without evidence of bladder contraction or detrusor overactivity [1]. Several factors contribute to the development of SUI, including vaginal delivery, aging, hysterectomy, recurrent urinary tract infections, smoking, the use of certain medications (e.g., diuretics), pelvic floor muscle dysfunction, and weight fluctuations [2]. Urinary incontinence affects up to 50% of women globally and nearly 77% of elderly women, significantly impairing quality of life through emotional distress, social withdrawal, and reduced self-esteem [3].

Treatment options for SUI encompass behavioral interventions, pharmacological therapy, and surgery. However, drug treatments are often associated with adverse effects, limiting their long-term use. Therefore, non-pharmacologic approaches, particularly those targeting pelvic floor function, are gaining attention as effective and well-tolerated alternatives [4].

Low-level laser therapy (LLT), also known as photobiomodulation therapy, has emerged as a novel, non-invasive modality used in various medical fields due to its regenerative and anti-inflammatory effects. LLT enhances mitochondrial activity by stimulating cytochrome C oxidase, leading to increased adenosine triphosphate (ATP) production, collagen synthesis, and cellular proliferation, thereby promoting tissue repair and analgesia [5][6].

Moreover, preliminary studies suggest that LLT may modulate local immune responses and improve microcirculation in pelvic tissues, potentially contributing to long-term symptom relief in patients with pelvic floor disorders.

In recent years, LLT has been applied in the management of several pelvic floor disorders, including vaginal atrophy, dysmenorrhea, chronic pelvic pain, and SUI [7].

Mousa et al. (2021) reported that LLT applied to pelvic floor muscles significantly reduced symptoms of SUI [8]. Similarly, De Marchi et al. demonstrated that combining LLT with pelvic exercises was more effective than exercise alone in improving continence [9]. Another study by da Silva et al. showed that transvaginal laser therapy enhanced pelvic muscle tone and responsiveness to training [10].

While previous studies have employed either transvaginal or perineal external laser applications, the present study aims to evaluate the safety and efficacy of a combined transvaginal and intra-bladder LLT protocol in perimenopausal women with stress urinary incontinence. This novel dual-approach method seeks to target deeper layers of the pelvic floor and bladder fascia to provide enhanced therapeutic outcomes.

## Case Presentation

The study included 12 perimenopausal women (aged 48-66 years) diagnosed with symptomatic stress urinary incontinence (SUI) who were referred to a gynecologist for treatment. A complete medical history was obtained from all participants, including data on previous pregnancies, mode of delivery, body mass index (BMI), history of constipation, alcohol and tobacco use, and general medical conditions. All patients had previously taken medications to alleviate symptoms and had practiced Kegel exercises, although adherence was inconsistent in many cases.

A vaginal examination was performed to assess for atrophy, and all patients underwent a cough stress test and measurement of post-void residual urine volume (PVR) using catheterization prior to initiating laser therapy. Patients were also asked to complete the Overactive Bladder Symptom Score (OABSS) and the Questionnaire for Urinary Incontinence Diagnosis (QUID). Additional evaluations included blood glucose testing, urinalysis, and ultrasound imaging of the kidneys, bladder, uterus, and ovaries.

Exclusion criteria included urinary calculi, prior urinary tract surgery, and the presence of large pelvic tumors (e.g., fibroids).

## Materials and Methods

**Figure-1:**
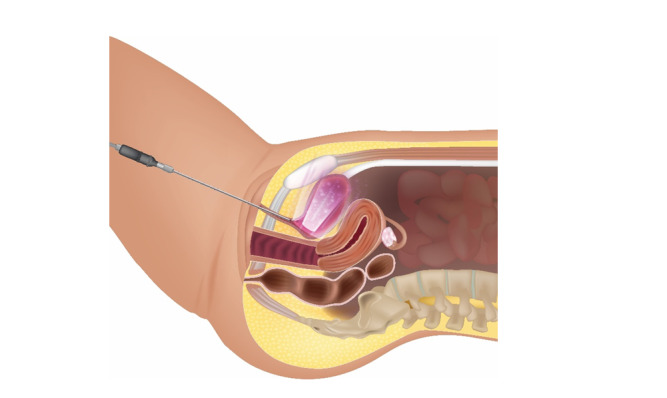
Figure 1. At the beginning of the treatment, the patients completely emptied their bladder, and the residual urine was removed using a Nelaton catheter. Then, 10 mL of normal saline was instilled into the bladder and immediately drained. A Medlight optical fiber, connected to a laser source with a wavelength of 808 nm, was inserted into the bladder through the catheter, and laser irradiation was applied for 8 minutes.

**Figure-2:**
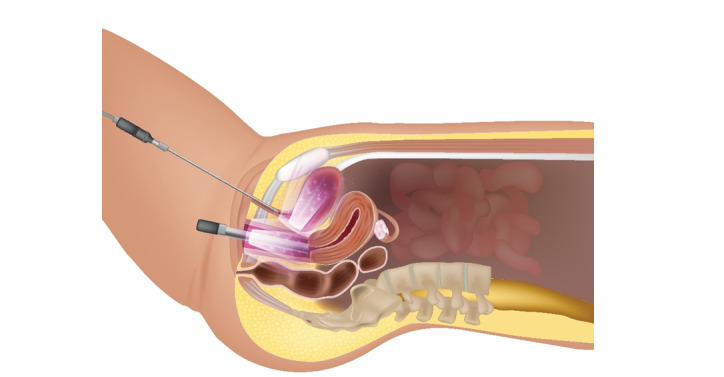
Figure 2. After removing the Medlight fiber, 30 mL of normal saline was re-injected into the bladder to enhance laser light scattering, and the Nelaton catheter was removed. The entire vaginal mucosa was then irradiated using a 360-degree vaginal applicator delivering simultaneous laser wavelengths of 630 nm and 780 nm, via a fiber combiner connected to the laser device. Simultaneously, an 808 nm Medlight fiber coated with lubricant gel was inserted into the urethra, and both the vagina and urethra were irradiated concurrently for 15 minutes.

**Figure-3:**
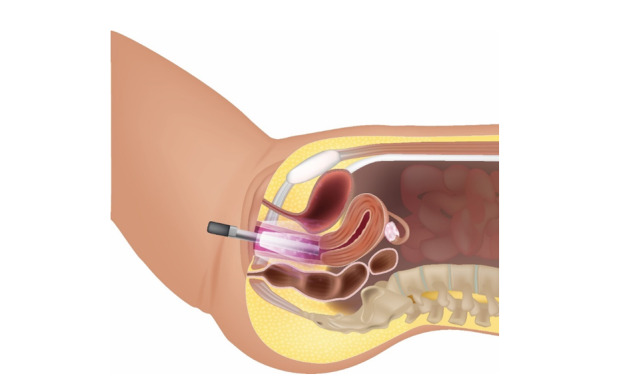
Figure 3. The Medlight fiber was removed from the urethra, and the vagina was further irradiated with 808 nm wavelength laser for an additional 5 minutes. During this phase, patients were instructed to contract their pelvic floor muscles 10 times, each for 6 seconds, followed by 6 seconds of relaxation, and then repeat the sequence after a short rest.

The low-level laser therapy protocol lasted for 12 weeks: twice weekly sessions in the first month, and once weekly sessions during the second and third months, totaling 16 treatment sessions. The OABSS and QUID assessments were administered both before the initiation of treatment and after its completion. The Overactive Bladder Symptom Score (OABSS) is a validated questionnaire comprising four items assessing urinary urgency, frequency, nocturia, and urgency incontinence. The total score ranges from 0 to 15, with higher scores indicating more severe symptoms. A reduction of ≥3 points is generally considered clinically significant. The Questionnaire for Urinary Incontinence Diagnosis (QUID) consists of six items evaluating the severity and frequency of stress and urge incontinence symptoms. Each item is scored from 0 to 5, with a maximum total score of 30. A ≥50% decrease in total score is considered clinically meaningful. Questionnaires were completed by the patients at baseline and at week 12, under supervision of the research nurse. To ensure consistency and minimize reporting bias, the same nurse administered all assessments throughout the study, and participants were instructed not to alter their lifestyle habits other than the prescribed exercises during the 12-week period.

This clinical trial was retrospectively registered in the Iranian Registry of Clinical Trials (IRCT) under the registration code IRCT20231203060255N2 on June 30, 2024.

### Procedure

As illustrated in Figure-1, patients first voided completely. Residual urine was then evacuated using a Nelaton catheter, followed by the instillation of 10 mL of saline into the bladder, which was promptly aspirated before initiating laser therapy. For intra-bladder laser irradiation, a cylindrical optical diffuser (model RD-ML, Medlight S.A., Switzerland) was used. The projection head, connected to a laser fiber and diode, was inserted into the bladder via the catheter.

The laser diode operated at 500 mW with a wavelength of 880 nm, and irradiation was performed for 8 minutes. Following this, the Medlight device was removed, and 30 mL of saline was reintroduced into the bladder to enhance laser light scattering. The catheter was then removed without draining the saline.

In the subsequent step, a 360-degree vaginal probe emitting at 630 and 780 nm was inserted into the vagina. Concurrently, a Medlight fiber with a wavelength of 808 nm was placed in the urethra. Both devices were simultaneously activated using a beam combiner fiber, with irradiation durations of 15 minutes for the urethra and 5 minutes for the vagina. The urethral fiber was then removed, and a second 5-minute vaginal irradiation was performed, as depicted in Figure-2. During laser irradiation, patients were instructed to perform pelvic floor contractions (10 repetitions of 6-second contractions followed by 6-second relaxations), repeated after a short break, for a total of 20 repetitions. Patients were also advised to perform 10-20 pelvic floor muscle training exercises twice weekly in three sets (approximately 15 minutes total) and to avoid any pharmacological treatments during the study period, as shown in Figure-3.

At the end of the treatment, participants were asked to rate their overall improvement on a scale from 1 to 10, with 1 indicating minimal improvement and 10 representing significant improvement.

### Statistical Analysis

At the end of the first and third months of treatment, patients were assessed for improvements in clinical symptoms. After four weeks, approximately 50% improvement in symptoms was reported by the participants. By the end of 12 weeks, the questionnaires were completed again, and the overall symptom improvement rate exceeded 80%. Notably, no vaginal infections or abnormal discharges were reported during the 12-week follow-up period. An additional benefit observed was an improvement in the quality and frequency of sexual intercourse, reported as a secondary outcome.

Continuous variables were expressed as mean ± standard deviation (SD), while categorical variables were reported as absolute numbers and percentages. The Shapiro-Wilk test was used to evaluate the normality of the data distribution. To compare pre- and post-treatment scores of the Overactive Bladder Symptom Score (OABSS) and the Questionnaire for Urinary Incontinence Diagnosis (QUID), the paired sample t-test was applied. A P-value of less than 0.05 was considered statistically significant. All statistical analyses were performed using SPSS version 18 (IBM Corp., Armonk, NY, USA) and GraphPad Prism version 7 (GraphPad Software Inc., CA, USA).

## Results

**Figure-4:**
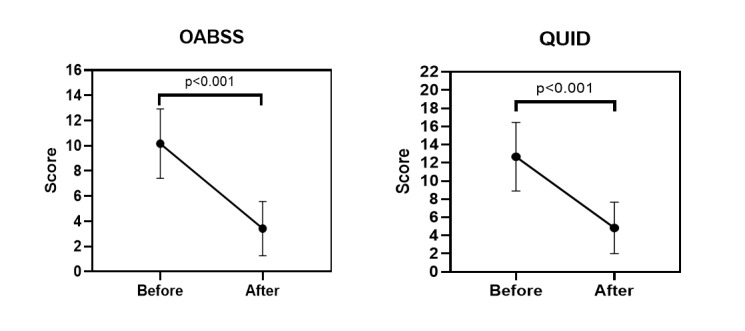
Figure 4. Column mean, error bars and mean connected for comparing OABSS and QUID for before and after of intervention

**Table:** Table1. Demographic Characteristics and Health Conditions of the Studied Population

**Continuous Variables**	**Mean (S.D)**
Age group (years)	54.3(5.9)
previous pregnancy	3.7(1.2)
previous NVD	2.6(1.0)
BMI	29.5(2.5)
FBS	97.6(8.7)
**Categorical variables**	**Number of cases (12)** **N (%) **
Past surgery	
No	3(25.0)
Yes	9(75.0)
Past history	
No	7(58.3)
Yes	5(41.7)
Past medication	
No	5(41.7)
Yes	7(58.3)
Constipation	
Negative	8(66.7)
Positive	4(33.3)
Stress test	
Negative	8(66.7)
Positive	4(33.3)
Vaginal atrophy	
Negative	7(58.3)
Positive	5(41.7)

**Table:** Table2. Comparison of Mean Scores of OABSS and QUID Before and After the Intervention

**Outcome**	**Before (Mean ± SD) **	**After (Mean ± SD) **	**p-value**	**Clinically Significant Change **
OABSS	10.17 ± 2.75	3.42 ± 2.15	<0.001*	Yes (≥3 points reduction)
QUID	12.67 ± 3.77	4.83 ± 2.82	<0.001*	Yes (≥50% reduction)

The demographic characteristics and health conditions of the studied population were shown in Table-1.

According to the results of paired samples t-test, there were statistically significant differences in both OABSS and QUID scores before and after the intervention (P<0.001 for both). The mean OABSS score decreased from 10.17 (SD 2.75) to 3.42 (SD 2.15), and the mean QUID score decreased from 12.67 (SD 3.77) to 4.83 (SD 2.82). This reduction corresponds to more than 60% improvement in symptom severity for both scales. Based on previous studies [REF], a reduction of ≥3 points in OABSS and ≥50% reduction in QUID score is considered clinically significant. Thus, the changes observed in our study reflect both statistically and clinically meaningful improvement (Table-2).

Figure-4 visually illustrates the mean scores and standard deviations of OABSS and QUID before and after the intervention, confirming the significant symptom improvement.

## Discussion

This case series demonstrates promising outcomes for low-level laser therapy (LLLT) as a non-invasive modality in the management of stress urinary incontinence (SUI) among perimenopausal and menopausal women. The combined application of transvaginal and intravesical laser stimulation, alongside structured pelvic floor muscle training (PFMT), led to notable improvements in urinary symptoms and overall quality of life.

Our findings are consistent with prior research suggesting that LLLT can enhance pelvic floor muscle tone and improve continence. For instance, Mousa et al. reported significant improvement in pelvic floor strength and urinary control following combined LLLT and PFMT in women with SUI [11]. Similarly, Gaspar and Brandi showed that non-ablative vaginal laser therapy improved continence scores and vaginal health without major adverse effects [12]. Importantly, no adverse events were observed during the intervention, aligning with previous reports highlighting the safety of LLLT in urogynecologic applications [13]. In addition, we noted improvements in sexual function, a secondary outcome that has also been observed in earlier trials investigating vaginal laser therapy [14].

Nevertheless, this study has limitations. The absence of a control group and the small sample size may limit generalizability. Furthermore, the retrospective trial registration and short follow-up duration restrict conclusions regarding long-term efficacy and safety. Larger randomized controlled trials are necessary to validate these preliminary findings and establish standardized LLLT protocols for SUI management.

## Conclusion

This study highlights the potential of low-level laser therapy (LLT) as a safe, non-invasive, and effective therapeutic option for stress urinary incontinence in perimenopausal and menopausal women. Given its favorable safety profile and additional benefits such as improved sexual function, LLT may serve as a valuable adjunct or alternative to conventional pharmacological and surgical interventions. Future well-designed clinical trials are essential to confirm these preliminary results and to establish standardized treatment protocols.

## Conflict of Interest

Authors do not have any conflict of interest or receive any fund. We did not receive any funding from any institution. The datasets used and/or analyzed during the current study are available from the corresponding author on reasonable request.
